# Why tibial plateau fractures are overlooked

**DOI:** 10.1186/s12891-018-2170-z

**Published:** 2018-07-21

**Authors:** Cecilie Mullerup Kiel, Kim Lyngby Mikkelsen, Michael Rindom Krogsgaard

**Affiliations:** 1 Section for Sports Traumatology M51, Bispebjerg-Frederiksberg Hospital (Part of IOC Research Center Copenhagen), University of Copenhagen, Bispebjerg Bakke 23, DK-2400 Copenhagen, NV Denmark; 2 Danish Patient Compensation Association, Kalvebod Brygge 45, 1560 Copenhagen, NV Denmark

**Keywords:** Tibial plateau fracture, Magnetic resonance imaging (MRI), X-rays, Pittsburgh knee rules, Clinical decision rules, Knee fracture

## Abstract

**Background:**

Tibial plateau fractures (TPFs) are sometimes overlooked in the emergency room (ER). Using a national register covering 18 years we aimed to find out why and to evaluate if use of a specific radiographic decision rule, Pittsburgh Knee Rules (PKRs), could have reduced the number of overlooked TPFs.

**Methods:**

Medical records for 137 patients, prospectively registered during 18 years by the Danish Patient Compensation Association (DPCA) (a national register), were studied. The inclusion criterion was a delayed diagnosis of a fracture in the knee following a trauma. Case records, legal assessments, and evaluations by specialist doctors were reviewed, and the consequences of the delayed diagnosis for outcome and treatment were registered.

**Results:**

Only 58 patients (42%) had been evaluated according to PKRs. In 53 patient cases, the fracture was not diagnosed on radiographs obtained at the first medical contact. However, in 84% of these cases, the fracture was visible or was suspected by retrospective evaluation. 50 out of 79 patients, for whom X-rays were not obtained, were candidates for radiographs according to PKRs, 17 cases lacked information to evaluate by PKRs and 12 cases were not candidates. In 53% of all cases, it was evaluated that the fracture position had worsened at the time of diagnosis. A significant disability compensation was granted in 36% of cases due to the delayed identification of fractures, totaling 841,000 EUR.

**Conclusions:**

The major reasons for overlooking TPFs were 1) difficulty in recognizing the fractures on X-rays and 2) that X-ray decision rules were not employed. Two thirds of the patients, for whom a radiograph had not been prescribed, would have had an X-ray, if the PKRs had been used. Overlooking TPFs significantly increased patient disability in one third of cases. We recommend that healthcare professionals in the ER use X-ray decision rules in addition to clinical examination to avoid overlooking TPFs. When standard radiographs are evaluated as normal in patients that are clinically suspect of a TPF, oblique X-rays, magnetic resonance imaging (MRI) or Computed Tomography (CT)-scan should be considered.

## Background

**Fig. 1:**
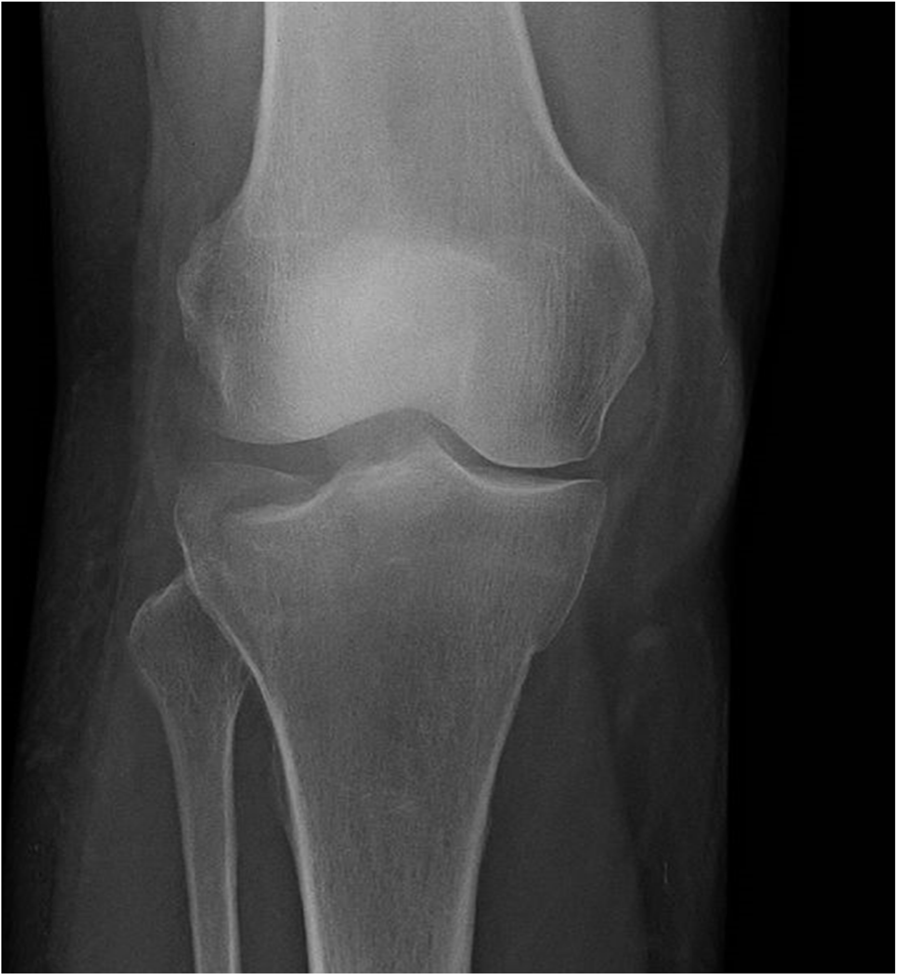
X-ray imaging of a lateral tibial plateau fracture. From https://commons.wikimedia.org/wiki/File:Tibial_Plateau_Fracture.jpg

Knee fractures account for about 6% of all trauma admissions to hospitals [1]. Tibial plateau fractures (TPFs, Fig. 1) comprise 1% of fractures in all age groups [2] and 8% in the elderly population [3]. The incidence of TPFs in Denmark is 10.3 per 100,000 annually [2]. The fractures can be difficult to recognize on standard radiographs [4], since the uninjured joint surface is often projected on top of the fractured part of the tibiae plateau in the anterior-posterior, and side views. TPFs are one of the most common radiological pathologies to be overlooked by doctors [5]. However, to the best of our knowledge there are only two studies addressing overlooked TPFs. The first [6] focused on overlooked fractures of the anterior tibial plateau, whereas the other [7] described eight overlooked osteoporotic fractures in elderly patients.

The Pittsburgh Knee Rules (PKRs) and similar decision-making rules were developed to improve accuracy in the use of X-ray imaging in cases of acute knee injuries. PKRs have a sensitivity close to 100% [8–13] and an inter-observer agreement of 0.71 [9]. Additionally, it has been demonstrated that the use of PKRs reduces the number of X-ray images obtained at hospitals by 30–78% [12, 14]. It is unknown if failure to obtain X-ray images at the primary healthcare contact for a knee injury is an underlying cause of overlooking TPFs. The objectives of this study were 1) to analyze why TPFs were overlooked in Denmark during an 18-year period, 2) to study the consequences of a delayed diagnosis for treatment and patient outcome, and 3) to evaluate if diagnosis of TPFs could have been established by use of PKRs.

## Methods

### Type of clinical study

This is a retrospective, descriptive study of prospectively collected cases of overlooked TPFs in Denmark 1996–2013 from the Danish Patient Compensation Association (DPCA). DPCA is a national public body, to which any patient or healthcare professional who experiences malpractice or complications in connection with treatment in the Danish healthcare system (private or public) can freely submit a compensation claim. Healthcare professionals are legally obligated to inform the patient about DPCA if this is suspected. The system is a “no fault” scheme, where no individual is blamed, and it focuses only on compensation. Information about the DPCA is described in [15], which also explains how claims are approved or rejected. A person’s disability is expressed in % according to a table from the National Board of Industrial Injuries in Denmark. “Additional disability” involves the extra disability (expressed in %) caused by treatment malpractice. Additional disability of < 5% is regarded as insignificant and is not compensated.

### Inclusion criterion

We included claims of delayed diagnosis of a fracture in the knee following a knee trauma (defined as a blunt trauma, a fall, a twist of the knee, or other injuries). The trauma had to be referable to an exact date to be included. Cases with a diagnostic delay of less than 24 h were excluded.

Clinical information on date of injury, first contact with medical care, date of correct diagnosis, trauma mechanism, patient age at the time of injury, diagnostic modalities used at first contact with medical care, side of injured knee, fracture classification according to ICD10 [16], treatment, outcome (expressed as disability %), and eventual monetary compensation for additional disability was manually collected from the included patients’ records.

### Decision rules

**Fig. 2:**
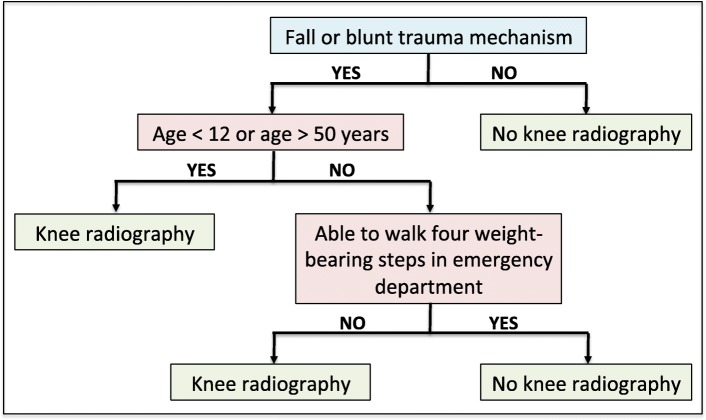
Flowchart of the Pittsburgh Knee Rules

Several decision rules exist to establish the indication for radiography of the knee. However, the PKRs were chosen in this study due to the high inter-observer agreement and sensitivity of these rules. The PKRs state that a radiograph of the knee is indicated following a fall or blunt trauma if the patient is younger than 12 or older than 50 years of age, or if the patient is unable to walk four weight-bearing steps (see flow chart of the decision rules in Fig. 2).

Hence, to evaluate the use of PKRs in our cases, the medical records were examined for patient age, evidence of trauma mechanism, and the patient’s ability to walk four weight-bearing steps at first medical contact. We defined that patients had not been able to walk four weight-bearing steps if this was specifically stated in the records, or if the patient was discharged with crutches.

The DPCA evaluation of the compensation claims included evaluation by orthopedic surgeons of the patient history and the clinical examination of the knee at the first medical contact. Moreover, it was evaluated whether the fracture was in fact visible on radiographs obtained at the first medical contact. Based on information about the following course, the orthopedic surgeon deemed if the fracture position had worsened during the period of diagnostic delay. At the time when no further change in the condition was expected, it was evaluated, based on specific medical examination, if the diagnostic delay had caused additional disability. Depressed fractures were regarded as displaced.

### Data handling

The use of personal data, including healthcare data, is protected by the Danish Act on Processing of Personal Data [15]. The study was therefore performed under the general permission for data collection to the Danish Patient Compensation Agency (2012–42-0325), and no further approval was necessary, as the data leaving the DPCA were anonymous. Ethical approval was not necessary, as the patients were not contacted.

## Results

A total of 156 cases met the inclusion criterion, but19 cases were excluded, leaving 137 cases for the study. The reasons for exclusion were double registration (9 cases), absence of trauma to the knee (7 cases), and that the fracture was not a TPF (one torus fracture, one patella fracture and one epiphysiolysis). Of the included cases, 64 cases (47%) involved fractures to the right knee, 72 cases (53%) to the left knee, and one case involved a patient with fractures in both knees. 65% of fractures were located in the lateral tibia condyle. Fracture displacement was seen in 78% of cases, 21% of fractures were not displaced, and in one fracture displacement could not be determined with certainty. Displacement ranged between 3 and 20 mm.

**Fig. 3:**
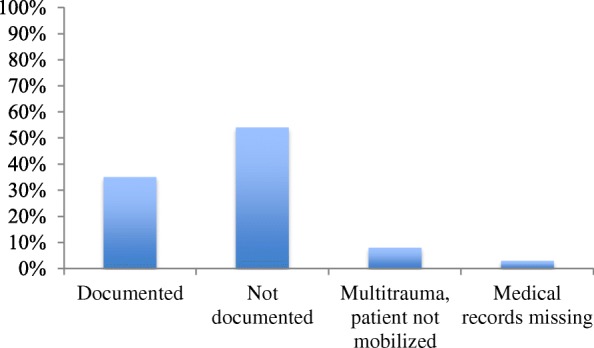
Bar chart showing the distribution of patients divided into four categories depending on whether or not weigh-bearing was documented, whether the patient suffered from multi-trauma preventing mobilization, and whether the medical record was missing relevant data

In 35% of the cases, it was documented if the patient could walk four weight-bearing steps at the first point of medical contact, while in 54% of cases, this was not documented. 8% of the patients could not be mobilized due to multi-trauma (see Fig. 3).

**Fig. 4:**
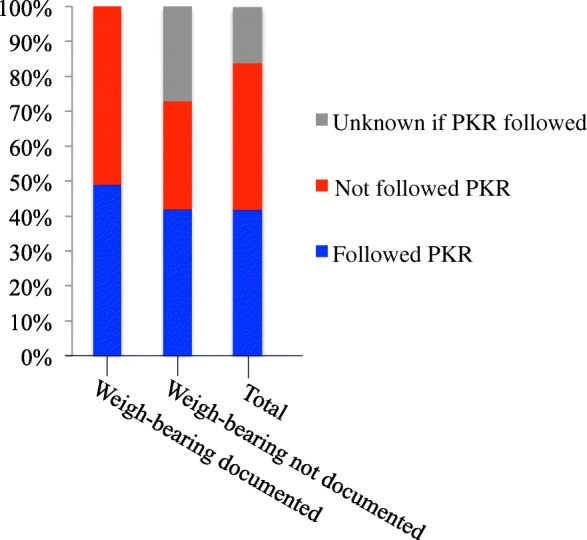
Bar chart showing whether PKRs had been followed or not and the correlation to documentation of weight-bearing ability

According to the information in the medical records, the PKRs had been followed in 42% of cases and had not been followed in 41% of the cases. In 17% there was not enough information to evaluate if PKRs had been followed. The correlation between whether PKRs had been followed and if weigh-bearing was documented in the patients file is shown in Fig. 4.

Fracture types according to ICD-10 are presented in Table 1.

**Table 1:** Details of the overlooked fractures. Seven patients had two diagnoses

Diagnose code ICD-10	Number of fractures
DS821 - Fractura partis proximalis tibiae	10
DS821A - Fractura condyli tibiae (Y-fracture)	3
DS821B - Fractura condyli tibiae lateralis	93
DS821C - Fractura condyli tibiae medialis	16
DS821D - Fractura eminentiae intercondyloideae tibiae	22

X-ray images were obtained for 53 patients (39%) at the first medical contact, and not obtained in 79 of cases (58%). In three cases there were radiographs from abroad, and in two cases radiographs were performed within 24 h after the initial medical contact. In 50 of the 79 un-imaged cases, X-ray imaging would have been indicated by the PKRs. In 12 cases the PKRs would not have indicated x-ray imagining and in 17 cases we do not have sufficient information to evaluate if PKRs would yield an X-ray examination or not. We estimate that if PKRs had been used in all cases, the number of overlooked knee fractures would have been reduced by 36%.

**Fig. 5:**
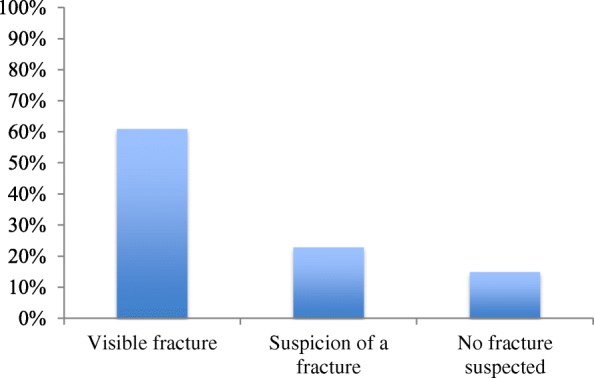
Bar chart showing whether a fracture was evaluated to be visible or not at retrospective evaluation

Thirteen of the undiagnosed fractures were discovered at radiological conference within a few days after the first medical contact. X-ray images had been obtained for four patients abroad, with fractures visible in two out of three cases, while the X-ray images were not available in the fourth case. Retrospective evaluation of all X-ray images available at first medical contact was performed at the DPCA by orthopedic specialists. As seen in Fig. 5, 61% of fractures were evaluated as visible, in 23% there was suspicion of a fracture, and in 15% of cases fractures were not visible or suspected. Original X-ray images were missing in one case.

The mechanism of trauma was blunt in 40% of cases and a fall in 49% of cases, and by other mechanisms in 11%. In 78% of the cases, it was a junior doctor or resident doctor who was the responsible healthcare providing individual. In one case, a radiologist was responsible, in 7% of cases general practitioners provided healthcare, and in 15% of cases, specialist doctors were responsible. The average delay of diagnosis was 75 days (range: 1–536). Eight patients had a delay of four days or less. In 53% of cases, it was evaluated that the fracture position had worsened as a consequence of the diagnostic delay. Overlooking the fracture at the primary medical contact resulted in no additional disability in 52% of the cases. In 13% of cases, the patient had less than 5% additional disability, whereas in 35% of cases, the additional disability was 5% or more. Only seven patients received monetary compensation for additional loss of work ability caused by the diagnostic delay. The total compensation was equivalent to 841,000 EUR.

Table 2 lists the modalities that were used to establish the diagnosis.

**Table 2:** Methods to establish the diagnosis

Diagnostic measure	N	%
X-rays	78	55.7
MR	44	30.7
CT	5	3.6
Knee arthroscopy	4	2.9
Knee arthroscopy + X-rays	3	2.1
X-rays + CT	3	2.1
X-rays + MRI	3	2.1
MRI + CT	1	0.7

In most cases, a new treatment was initiated after the fracture had been diagnosed (see Table 3).

**Table 3:** Treatment after the fracture had been diagnosed

Treatment changed	80%
Surgical treatment optimal, but too late to perform	6%
No change of treatment	13%
Patient refused suggested change in treatment	1%

## Discussion

The major underlying reasons for undiagnosed TPFs included difficulty in recognizing the fractures on X-rays and that radiographs were not taken because PKRs (or similar rules) were not employed at the first medical contact. In 84% of the cases, where an X-ray image was obtained in the ER at first medical contact, the fracture was visible or suspected at retrospective evaluation of the X-rays. Anterior tibial plateau fractures can be hard to recognize in anterior-posterior as well as lateral projections [6]. TPFs can be overlooked on normal X-ray images due to the anatomy of the tibial plateau, which causes a distortion in radiography, underestimating anterior surface depression and overestimating posterior surface depression [17]. Osteoporotic fractures of the tibial plateau can be misdiagnosed, because primary X-ray images often look normal for 2–3 weeks, until an area of sclerosis is formed by collapse of cancellous bone in the tibial plateau [7]. It also affects the diagnostic sensitivity how the patient is positioned, since the only evidence of a TPF can be the presence of a fat/fluid level in the suprapatellar recess in the lateral projection, provided that the patient is positioned horizontally [4, 17]. Oblique projections should be considered in addition to standard projections when TPFs are suspected but not confirmed by standard AP- and lateral X-rays, as adding oblique knee views increases the sensitivity from 79 to 85% [18].

MRI-scan is considered the state-of-the-art for detection of occult fractures [19], and this or a CT-scan should be considered in patient cases with a clinical suspicion of a TPF and where X-ray images are normal even though CT-scans are fast and provide a detailed view of the fracture pattern and articular surface [4], MRI-scans are better suited for accurately displaying occult fractures and for visualizing soft tissue injuries, which often occur along with the fractures [20].

We found that for 50 patients an X-ray would have been indicated if PKRs had been followed. Even though TPFs can be difficult to identify on standard X-ray images, most of these fractures would likely have been discovered at the first medical contact, if the principles of the PKRs had been followed (and radiographs been obtained) in addition to the clinical examination in the ER setting. Figures from the Danish National Patient Register (extracted from the register’s website) show that between 2005 and 2013 a total of 8797 patients were discharged with a fracture of the proximal tibiae. Of these, 1489 were coded as tibial plateau fractures, and of the 7308 fractures that did not have a detailed coding, we estimate that half were TPFs. This entails that about 5000 TPFs occurred in Denmark during these 9 years. A study on the epidemiology of TPFs from Aalborg University hospital in Denmark supports this conclusion as it evaluated the incidence of TPFs to be 10.3 per 100,000 citizens annually [2]. The dark number (patients who have not claimed a case at DCPA) is unknown, but it is assessed that only 15–85% of all patients eligible for compensation, claim their case [15]. Overlooking a fracture is regarded as a grave mistake among patients, and we have estimated the dark number to be 100% of the registered cases in our study. From this it can be estimated that 3.9% of all TPFs in Denmark were not diagnosed at first medical contact. An implication of this finding may thus be that decision rules (such as PKRs) should be mandatory to employ in addition to clinical examination to triage knee trauma patients correctly. Additionally, these decision rules may also help minimize the number of X-ray images to be captured in the ER by identifying clinically relevant cases for X-ray examination [8, 9, 11–14, 21]. Not only would this save personnel time and ER resources, but it would also lower the overall patient exposure to X-rays. The finding that 12 patients (15% of the patients for whom an X-ray image was not obtained) would not have been offered X-ray imaging, if PKRs had been used, could be interpreted as low sensitivity of PKRs. However, in all of these 12 cases the trauma mechanism was not a blunt trauma or a fall, but in most cases a twist, and in principle the PKRs do not apply with these trauma mechanisms. In addition, these 12 patients were a very selective group, as they were not clinically suspected to have a fracture. Therefore, our findings do not conflict with the generally accepted 94–100% sensitivity of PKRs to identify TPFs [14].

In 7% of the examined cases, it was evaluated that surgical intervention would have been indicated, if fractures had been discovered at the first medical contact, which in most cases most likely would have resulted in a superior outcome. In 80% of patients the treatment was changed following diagnosis of their fracture, and many of these patients would likely have had a shorter rehabilitation period, if the fracture had been diagnosed at first medical contact. The finding that 36% of the patients in our study had an additional disability of 5% or more because the primary diagnosis of their fracture was missed, underlines that overlooking a TPF has a significant negative effect on patient outcome.

## Limitations to the study

A limitation of this study is that the specialist consultants who performed the retrospective viewing of the X-ray images in most cases knew what they were looking for, which might have interfered with their evaluation of whether a fracture could have been diagnosed at first medical contact or not. Also, our study underestimates the true incidence of overlooked TPFs, since only patients who file a claim are registered in the DPCA database, as addressed in our discussion.

## Conclusion

The most important underlying reasons for overlooking TPFs in the ER setting of Danish hospitals were 1) that TPFs were not identified on X-rays and 2) X-rays were not taken, because decision rules for indication of knee X-rays had not been followed in the ER setting. If a fracture is clinically suspected in case of normal AP and lateral X-rays, oblique projections should be considered. However, MRI or CT-scans are more sensitive and should be performed if clinical suspicion persists. To prevent a potential fracture from displacement, the patient should be immobilized with a fixed knee brace and disabled from bearing weight until a final diagnosis has been reached.

We estimated that if PKRs had been used in all patients, the number of overlooked knee fractures would have been reduced by 36%. As overlooking a TPF often results in additional disability for the patient, it would be beneficial if healthcare staff in the ERs used clinical decision rules in addition to the clinical examination. This holds the potential to save personnel time, hospital resources, and lower the overall patient exposure to X-rays.

## Abbreviations

CT: Computed Tomography
DPCA: Danish Patient Compensation Association
ER: Emergency Room
MRI: Magnetic Resonance Imaging
PKRs: Pittsburgh Knee Rules
TPF: Tibia Plateau Fracture
